# A novel mutation in *VPS33B* gene causing a milder ARC syndrome phenotype with prolonged survival

**DOI:** 10.1002/jmd2.12027

**Published:** 2019-03-22

**Authors:** Rodrigo del Brío Castillo, James E. Squires, Patrick J. McKiernan

**Affiliations:** ^1^ Pediatric Liver Service Hospital Infantil Universitario La Paz Madrid Spain; ^2^ Pediatric Hepatology Children's Hospital of Pittsburgh of UPMC, University of Pittsburgh Medical Center Pittsburgh Pennsylvania

**Keywords:** arthrogryposis‐renal dysfunction‐cholestasis, neonatal cholestasis, prolonged survival, *VIPAS39*, *VPS33B*

## Abstract

**Introduction:**

ARC (arthrogryposis, renal dysfunction, and cholestasis) syndrome is an uncommon multisystem disorder that entails a very poor prognosis. It is caused by mutations in either *VPS33B* or *VIPAS39* gene, both playing a key role in intracellular trafficking. We report two siblings born to first cousin parents with a novel mutation in *VPS33B* who have both shown prolonged survival.

**Cases Presentation:**

The index patient presented with bilateral hip dysplasia and arthrogryposis, failure to thrive, undernourishment, developmental delay, and low gamma‐glutamyl transferase cholestasis. She at age 2 years underwent external biliary diversion with improvement in pruritus but liver disease continued to progress. She developed stomal bleeding at 7 years of age and liver biopsy displayed cirrhosis. Her 3‐year‐old sibling showed a similar trajectory as well as he had ichthyotic skin with excoriations. Their renal involvement was mild and stable. Genetic analysis in both patients revealed a novel homozygous mutation in NM_018668.4 (*VPS33B*):c.1157A > C (p.His386Pro).

**Conclusions:**

ARC syndrome is a severe disorder with few patients reported to survive beyond 12 months of age. This report discloses a novel mutation in the *VPS33B* gene and describes a phenotype with prolonged survival, mild renal involvement, and progressive liver disease.

**Synopsis**

ARC syndrome, a heterogeneous multisystem disorder usually fatal in infancy, is caused by mutations in either *VPS33B* or *VIPAS39* gene. This report expands the phenotype by describing prolonged survival associated with a novel mutation in the *VPS33B* gene.

## 1 INTRODUCTION

Arthrogryposis‐renal dysfunction‐cholestasis (ARC) syndrome (OMIM #208085 and #613404) is an uncommon autosomal recessive disorder that was first described in two siblings born to consanguineous parents.^1^ Liver involvement classically presents as low gamma‐glutamyl transferase (GGT) cholestasis with mildly elevated alanine aminotransferase (ALT)/aspartate aminotransferase (AST), and histology may show giant cell transformation of hepatocytes, bile duct paucity, and lipofuscin deposition. Renal disease ranges from renal tubular acidosis to Fanconi syndrome to nephrogenic diabetes insipidus. Arthrogryposis was originally described as neurogenic although abnormal collagen formation in different tissues may play a role.^2^ As additional features have been described, it has become known that the phenotype is variable.^3^ Severe failure to thrive and ichthyosis are characteristic and other features include dysmorphic appearance, corpus callosum dysgenesis, deafness, and platelet dysfunction.^4^, ^5^ No curative treatment exists for ARC syndrome and prognosis remains very poor, with few patients surviving beyond 12 months of age.^4^, ^6^ Leading mortality factors in these patients are dehydration, infections, and bleeding disorders.^4^, ^5^

ARC syndrome is caused by mutations in either *VPS33B* (vacuolar protein sorting 33B yeast homolog) or *VIPAS39* (also known as VIPAR‐VPS33B interacting protein, apical‐basolateral polarity regulator) gene.^7^, ^8^ These play an essential role in intracellular vesicle transport, membrane protein trafficking, and maintenance of cell polarity in many tissues, explaining its multisystemic involvement.^9^ Interactions between the VPS33B‐VIPAR complex and RAB11A are responsible for trafficking canalicular proteins such as BSEP and CEA via the apical recycling endosome in hepatocytes; therefore, these proteins are mislocalized in ARC syndrome patients.^10^, ^11^ In addition, the VPS33B‐VIPAR complex participates in the development and maturation of platelet α‐granules, which is required to form stable aggregates.^12^, ^13^ VPS33B‐VIPAR deficiency leads to abnormal morphology of epidermal lamellar bodies, which affects epidermal homeostasis and disrupts skin barrier function.^9^, ^14^

We report two siblings with a novel mutation in the *VPS33B* gene who have both shown prolonged survival.

## 2 CASES PRESENTATION

The index patient was born to first cousin Arabian parents at term after a normal pregnancy weighing 2.5 kg. She was noted to have bilateral hip dysplasia, and developed failure to thrive, low GGT cholestasis, and developmental delay. She underwent external biliary diversion at 2 years of age with improvement in pruritus. She remained undernourished despite continuous overnight feeding. She demonstrated global developmental delay, being able to sit independently at 11 months old, and her first words were at 4 years of age. She underwent bilateral femoral osteotomies and open reduction of the left hip at 4 years of age following which she walked for the first time. She attends a special needs school on a full‐time basis. She developed recurrent stomal bleeding at 7 years of age requiring blood transfusion, which eventually responded to oral propranolol. A liver biopsy at that time showed established cirrhosis.

When evaluated at 7.7 years of age, she was undernourished with short stature. She showed limited elbow extension and hip abduction, dry skin, and finger clubbing. Abdominal examination revealed hepatosplenomegaly. Neurological examination showed generalized hypotonia and reduced muscle bulk and strength. Further investigations are summarized in Table 1.

**Table 1:** Summary of patient characteristics

	Index patient	Younger sibling
Age	7.7 years old	3 years old
Growth/Nutrition	Undernourishment despite continuous overnight feeding Short stature	Undernourishment with short stature
Skeletal involvement	Arthrogryposis Bilateral hip dysplasia	Arthrogryposis
Renal involvement	Normal TRP and GFR Albumin/creatinine ratio 65.2 mg/g (N < 37) Mild aminoaciduria US: normal kidneys	Normal TRP and GFR Albumin/creatinine ratio 114.3 mg/g (N < 37) Mild aminoaciduria US: normal kidneys
Liver disease	Low GGT cholestasis and pruritus External biliary diversion at 2 years of age Established cirrhosis ALT 183 IU/L, AST 120 IU/L, GGT 82 IU/L Platelet count: 81000/L US: coarse liver appearance, elevated HA‐RI and splenomegaly	Low GGT cholestasis and pruritus Hepatomegaly ALT 309 IU/L, AST 205 IU/L, GGT 31 IU/L Platelet count: 268000/L US: coarse liver appearance otherwise normal
Neurodevelopmental involvement	Global developmental delay Hypotonia and reduced muscle bulk and strength Special needs school MRI: marked hypoplasia of corpus callosum, decrease in white matter volume, increased T1 signal in basal ganglia	Global developmental delay Hypotonia and inability to sit independently MRI: thin and hypoplastic corpus callosum, white matter hypoplasia and delayed myelination, increased signal in basal ganglia
Skin involvement	Dry skin	Ichthyosis and scratch marks

Abbreviations: ALT, alanine aminotransferase; AST, aspartate aminotransferase; GFR, glomerular filtration rate; GGT, gamma‐glutamyl transferase; HA‐IR, hepatic artery resistive index; IU, international units; MRI, magnetic resonance imaging; TRP, tubular reabsorption of phosphate; US, ultrasound.

Genetic analysis revealed a novel homozygous mutation in *VPS33B*:NG_012162.1 (NM_018668.4):c.1157A > C (p.His386Pro).

The second patient was the first patient's younger sibling. He was also born after a normal pregnancy and his birth weight was 4 kg. His initial clinical course showed a similar trajectory to his sister. He showed significant failure to thrive and developmental delay.

He was reviewed at age 3 years along with his sister. He was malnourished and had ichthyotic skin with scratch marks. He showed significant development delay with hypotonia and could not sit independently. He had limited extension of both elbows and knees, and limited abduction of the hips. Abdominal examination revealed hepatomegaly. Further investigations are summarized in Table 1.

Genetic testing revealed the same homozygous *VPS33B* mutation as his sister.

## 3 DISCUSSION

ARC syndrome is a rare multisystemic disorder characterized by arthrogryposis, renal tubular dysfunction, and low serum GGT cholestasis caused by mutations in *VPS33B* or *VIPAS39*.^7^, ^8^ Evidence of multiorgan involvement is common, in agreement with the ubiquitous location of VPS33B‐VIPAR. Although ARC syndrome is severe and usually fatal, there is clinical variability both in the number of affected organs and in the degree of dysfunction. Most children have a very poor prognosis with death in the first year of life due to fluid loss, infections, and bleeding.^4^, ^5^ However, occasional reports have showed prolonged survival.^4^, ^15^, ^16^, ^17^, ^18^

We present two siblings homozygous for a novel mutation in NM_018668.4 (*VPS33B*):c.1157A > C (p.His386Pro) who have survived to 7.7 and 3 years, respectively. Their clinical similarity is noteworthy, which has not always been the case in previously reported sibships.^4^ They both manifest arthrogryposis, neonatal low GGT cholestasis with progression to chronic liver disease, subclinical renal involvement, failure to thrive, and severe developmental delay with similar brain magnetic resonance imaging findings. Although this mutation has not previously been identified as pathogenic, functional prediction scores such as PolyPhen‐2 and CADD suggest that it is likely to be pathogenic. All cardinal features of ARC syndrome are present and comprehensive diagnostic testing has not revealed an alternative etiology. This missense variant is located in exon 15, resulting in a different amino acid sequence with preserved length. Numerous pathogenic mutations have been reported throughout the *VPS33B* gene, which suggests no obvious mutational hotspots.^17^

The only other mutation to date associated with prolonged survival is *VPS33B* c.1225 + 5G > C, which has been reported as a compound heterozygote in four children (mainly of Latin American descendancy). The first patient, who was 5.5 years old and also carried the deletion c.240‐577_290‐156del, presented with bilateral talipes, aminoaciduria and proteinuria, ichthyosis, cholestasis with pruritus that improved after cutaneous biliary diversion, failure to thrive with steady growth after gastric tube placement, developmental delay, and sensorineural hearing loss.^17^ A second patient that harbored the mutation c.1261_1262delCA was found to have arthrogryposis after birth, renal tubular dysfunction, mild cholestasis, and pruritus, and was reported to have undergone corrective surgery for hip dysplasia at 3 years old.^17^ A girl whose other mutation was c.1609_1657 + 9del died due to line‐associated sepsis at 8.5 years old.^18^ Finally, another girl with the mutation c.440_499del showed Fanconi syndrome, cholestasis, and pruritus who responded to enterobiliary anastomosis undertaken at age 6 years.^16^ Interestingly, even though the *VPS33B* c.1225 + 5G > C variant may depict a milder phenotype consistently, there is considerable clinical heterogeneity.

Prolonged survival raises the question of how best to manage systemic complications. Both patients we report have evidence of progressive liver disease and established cirrhosis with portal hypertension. The older child underwent external biliary diversion that seemed to have a positive effect on pruritus, although the underlying liver disease still appears progressive. A positive outcome from biliary diversion with relief from pruritus has been previously reported in patients with long survival.^16^, ^17^ Low density lipoprotein (LDL)‐apheresis has also been reported to lower serum bile acids with a reduction in pruritus and improved skin conditions in a single patient.^16^, ^19^

There is a single case report of a 12‐year‐old boy who underwent living donor liver transplantation for intractable pruritus. His pruritus responded immediately, and he showed significant nutritional catch up and remained well up to 5 years posttransplant when reported.^20^ Although his clinical features were in agreement with ARC, his diagnosis was not genetically confirmed. However, in the setting of progressive liver disease and stable extrahepatic disease, liver transplantation may be an option for some affected children.

In conclusion, we report two siblings affected by ARC with a novel mutation who have shown a very similar trajectory and prolonged survival. However, this remains a very severe disease with their quality of life being significantly impacted by liver disease, severe developmental delay, and orthopedic abnormalities. Counseling for families with this syndrome should include the possibility of prolonged survival.

## CONFLICT OF INTEREST

Rodrigo del Brío Castillo, James E Squires, and Patrick McKiernan declare that they have no conflict of interest.

## AUTHOR CONTRIBUTIONS

Rodrigo del Brío Castillo contributed to the literature review and wrote the manuscript. James E. Squires critically revised the article. Patrick McKiernan was responsible for patients' care and conceived and supervised the report. All authors have read and approved the final manuscript.

## DETAILS OF ETHICS APPROVAL AND PATIENT CONSENT STATEMENT

All procedures followed were in accordance with the ethical standards of the responsible committee on human experimentation (institutional and national) and with the Helsinki Declaration of 1975, as revised in 2000(5). Informed consent was obtained from all patients for being included in the study. This article does not contain any studies with animal subjects performed by any of the authors.
